# Promising cytotoxic activity profile of fermented wheat germ extract (Avemar^®^) in human cancer cell lines

**DOI:** 10.1186/1756-9966-30-42

**Published:** 2011-04-16

**Authors:** Thomas Mueller, Karin Jordan, Wieland Voigt

**Affiliations:** 1 University of Halle, Department Internal Medicine, Oncology/Hematology and Hemostaseology, Ernst-Grube Str. 40, 06120 Halle/Saale, Germany

## Abstract

Fermented wheat germ extract (FWGE) is currently used as nutrition supplement for cancer patients. Limited recent data suggest antiproliferative, antimetastatic and immunological effects which were at least in part exerted by two quinones, 2-methoxy benzoquinone and 2,6-dimethoxybenzquinone as ingredients of FWGE. These activity data prompted us to further evaluate the in vitro antiproliferative activity of FWGE alone or in combination with the commonly used cytotoxic drugs 5-FU, oxaliplatin or irinotecan in a broad spectrum of human tumor cell lines. We used the sulforhodamine B assay to determine dose response relationships and IC_50_-values were calculated using the Hill equation. Drug interaction of simultaneous and sequential drug exposure was estimated using the model of Drewinko and potential clinical activity was assessed by the model of relative antitumor activity (RAA). Apoptosis was detected by DNA gel electrophoresis.

FWGE induced apoptosis and exerted significant antitumor activity in a broad spectrum of 32 human cancer cell lines. The highest activity was found in neuroblastoma cell lines with an average IC_50 _of 0.042 mg/ml. Furthermore, IC_50_-range was very narrow ranging from 0.3 mg/ml to 0.54 mg/ml in 8 colon cancer cell lines. At combination experiments in colon cancer cell lines when FWGE was simultaneously applied with either 5-FU, oxaliplatin or irinotecan we observed additive to synergistic drug interaction, particularly for 5-FU. At sequential drug exposure with 5-FU and FWGE the observed synergism was abolished.

Taken together, FWGE exerts significant antitumor activity in our tumor model. Simultaneous drug exposure with FWGE and 5-FU, oxaliplatin or irinotecan yielded in additive to synergistic drug interaction. However, sequential drug exposure of 5-FU and FWGE in colon cancer cell lines appeared to be schedule-dependent (5-FU may precede FWGE).

Further evaluation of FWGE as a candidate for clinical combination drug regimens appeared to be warranted.

## Introduction

The exact chemical composition of FWGE, which is currently used as nutriment for cancer patients is not completely known [1]. It contains two quinones, 2-methoxy benzoquinone and 2,6-dimethoxybenzquinone that likely play a significant role in exerting several of its biological properties [2]. Preclinical in vitro and in vivo data suggested antiproliferative, antimetastatic and immunological effects of FWGE [1-7]. In cell lines studies, FWGE induced programmed cell death via the caspase - PARP-pathway [7,8]. But the exact mechanism by which this multi-molecule composition triggers cell death is still obscure. In previous studies several groups could demonstrate that FWGE interferes with enzymes of the anaerobic glycolisis and pentose cycle [2,9,10]. Known targets are the transketolase, glucose-6-phosphate dehydrogenase, lactate dehydrogenase and hexokinase which are necessary for the allocation of precursors for DNA-synthesis [9]. Also involved in DNA-synthesis is ribonucleotide reductase [6]. This enzyme is upregulated in various types of cancer and is an attractive target in cancer chemotherapy. Several established anticancer drugs like fludarabine, cytarabine and gemcitabine exert at least in part their cytotoxic activity by inhibiting ribonucleotide reductase [11]. An inhibitory activity on ribonucleotide reductase could also be demonstrated for FWGE, allowing FWGE to interfere with nucleic acid-synthesis by several pathways [1,8,11].

Beside the single agent cytotoxic activity of FWGE against human tumor cell lines and human tumor xenografts some data suggest synergistic drug interaction between 5-FU or DTIC in a limited number of cell lines [2,6].

In addition to the preclinical data there are already a few clinical studies published which suggest some beneficial effect of FWGE in human cancer therapy. The most impressive data were generated in a randomized Phase II trial by Demidov et al. who observed a significant gain in progression free survival and overall survival for the combination of DTIC and FWGE as compared to DTIC alone in melanoma patients [12]. A study conducted by Jakab et al. in patients with colorectal cancer found an enhanced survival and reduced metastasis formation for the combination of chemotherapy and FWGE as compared to chemotherapy alone group. In a multivariate analysis of this study only tumor stage and FWGE treatment were the only significant predictors of survival [13]. However, this data have to be interpreted with caution since the study had a non randomized design and the patient groups were not balanced [1,13]. Of similar importance, several studies including the ones cited above suggested an improvement of quality of life due to co treatment with FWGE [14].

Overall, the limited preclinical and clinical data available suggest some promising activity profile of FWGE as a nutriment for cancer patients but also a potential anticancer agent.

In this broad in vitro study we aimed to analyze the single agent activity of FWGE as well as its interaction with the commonly used drugs 5-FU, oxaliplatin and irinotecan in a large panel of human cancer cell lines from different tumor entities. These data are of potential value to direct the further development FWGE in different cancer types and to help to select potential drug partners for the future development of combinations of chemotherapy regimens with FWGE.

## Materials and methods

### Drugs and chemicals

FWGE was a generous gift from Biropharma Ltd, Kunfeherto, Hungary. FWGE was stored as dried powder at 4°C until use. For experimentation, FWGE was freshly prepared in sterile water to a final concentration of 100 mg/ml. After solution FWGE was centrifuged with 150 g to remove the insoluble material. 5-FU, Irinotecan, Oxaliplatin and Sulforhodamine B were purchased from Sigma Chemical Company, Germany. RPMI 1640 and Penicillin/Streptomycin were obtained from PAA, Pasching, Austria. FBS was purchased Biochrom AG, Berlin, Germany.

### Cell lines and culture

The following human cancer cell lines were used for experimentation: testicular cancer (H12.1, 2102EP, 1411HP, 1777NRpmet), colon cancer (HCT-8, HCT-15, HCT-116, HT-29, DLD-1, SW480, COLO205, COLO320DM), NSCLC (A549, A427, H322, H358), head and neck cancer (FADU, A253), cervical epidermoid carcinoma (A431), mammary adenocarcinoma (MCF-7, BT474), ovarian adenocarcinoma (A2780), gastric cancer (M2), anaplastic thyroid cancer (8505C, SW1736), papillary thyroid cancer (BCPAP), follicular thyroid cancer (FTC133), melanoma (518A2), hepatoma (HepG2), glioblastoma (U87MG), neuroblastoma (SHSY5Y, SIMA). All cell lines were grown as monolayers of up to 80% confluence in RPMI 1640 supplemented with 10% FBS and 1% Penicillin/Streptomycin at 37°C, 5% CO_2 _and humidified air.

### Growth inhibition experiments

To assess antiproliferative effects, the total protein sulforhodamine B (SRB) assay was used as described previously [15]. In brief, cells were seeded in 96 well plates at a cell line specific density to ensure exponential growth throughout the whole period of the assay. These cell numbers were determined previously by cell growth kinetics. After 24 h, exponentially growing cells were exposed to serial dilutions of each drug alone or drug combinations for the indicated times continuously. To investigate the influence of drug schedules drug A was added 24 h after cell seeding followed by drug B another 24 h later or vice versa. Corresponding control plates with single agents were treated in parallel.

After 120 h total assay time, media was removed and cells were fixed with 10% TCA and processed according to the published SRB assay protocol [15]. Absorbency was measured at 570 nm using a 96-well plate reader (Rainbow, SLT, Germany).

### DNA gel electrophoresis

To detect apoptosis by DNA gel electrophoresis the floating cells after drug treatment with an IC_90 _of FWGE for 48 h were used. After washing cells twice with PBS they were lysed in lysis-buffer (100 mM TRIS-HCL (pH8.0), 20 mM EDTA, 0,8% SDS). Subsequent to treatment with RNaseA for 2 h at 37°C and proteinase K (Roche Molecular Biochemicals) overnight at 50°C, lysastes were mixed with DNA loading buffer. To separate DNA fragments, probes were run on a 1.5% agarose gel followed by ethidium bromide staining and rinsing with destilled water. DNA ladders were visualized under UV light and documented on a BioDocAnalyse instrument (Biometra).

### Data analysis

Dose response curves were generated by Sigma Plot (Jandel Scientific, San Rafael, CA) and IC_50 _values were calculated based on the Hill equation. Drug interaction was assessed using the model of Drewinko [16]. In brief, a hypothetical curve was calculated by multiplying the ratio of treated and untreated control with the dose response data points of the single drug curve. Synergy could be assumed if the hypothetical curve runs above the combination curve and antagonism is indicated if the hypothetical curve runs below the combination curve. In case of additivity both curve were superimposed.

Statistical significance was probed with the two tailed, unpaired student's t-test. Significance was assumed at a p-value < 0.05.

Potential clinical activity was estimated by relative antitumor activity (RAA), which was defined as the ratio of peak plasma level and in vitro IC_50 _value [17]. A RAA > 1 indicates potential clinical activity.

## Results

### Single agent antiproliferative activity of FWGE in human cancer cell lines

**Figure 1:**
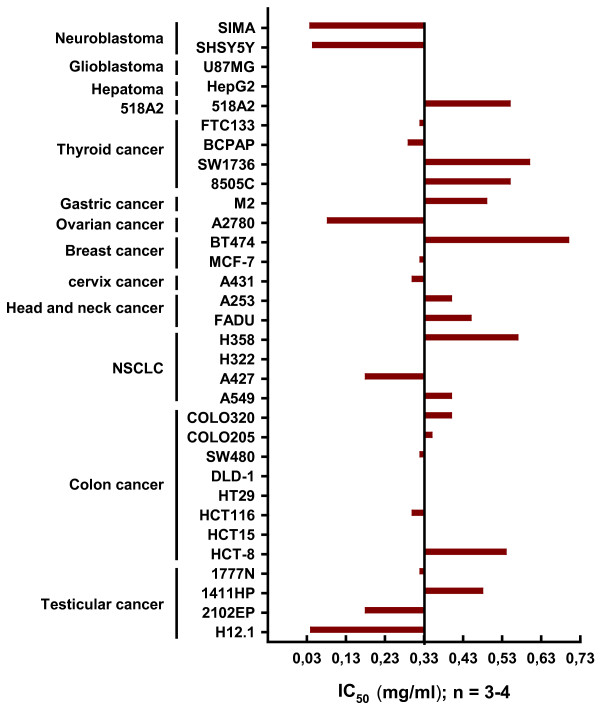
**Illustration of IC_50 _of FWGE as a mean graph**. IC_50 _of at least 3 independent experiments per cell line were averaged and summarized as a mean graph for better comparison of the different activity. The average IC_50 _is 0.33 mg/ml. The highest activity of FWGE was found on neuroblastoma and ovarian cancer cell lines. It's interesting to note that the IC_50_-values of the 8 human CRC cell lines included in this screen range close to the average IC_50_.

The antiproliferative activity of a 96 hour continuous exposure to FWGE was evaluated in a large panel of human tumor cell lines using the SRB-assay. IC_50_-values were calculated using the Hill equation and the obtained data from at least three independent experiments were summarized as a mean graph (Figure 1). IC_50 _of FWGE ranged from 0.038 mg/ml to 0.7 mg/ml with a median IC_50 _of 0.33 mg/ml.

Notably, the estimated peak plasma concentration after the oral intake of a standard dose of 9 g/day FWGE in patients is 0.5-1 mg/ml [7]. Considering this peak plasma concentration and the observed IC_50 _in our cell line screen, the calculated RAA is at least 1 or higher which could indicate potential clinical activity. The highest activity of FWGE was found in neuroblastoma cell lines with an average IC_50 _of 0.042 mg/ml (RAA ≈ 12-24). Of note, the 8 colon cancer cell lines included in this screen had a very narrow IC_50 _range varying from 0.3 mg/ml to 0.54 mg/ml yielding in a RAA of 1.7-3.3 (Figure 1).

### Detection of the mode of cell death induced by FWGE in a panel of cell lines

**Figure 2:**
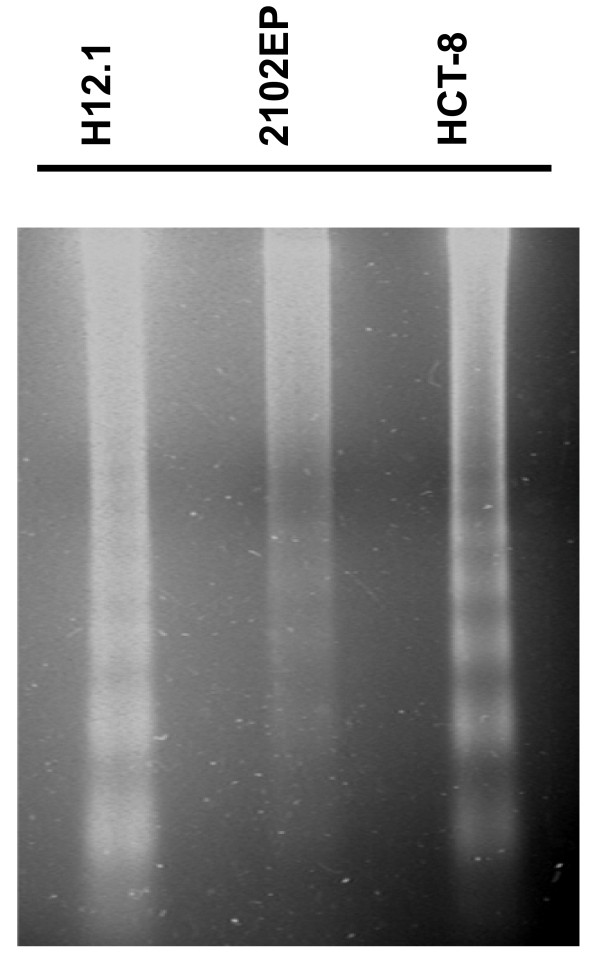
**Induction of apoptosis by FWGE**. A representative panel of human tumor cell lines was treated with an IC_90 _of FWGE for 48 h and floating cells were harvested by centrifugation for DNA extraction. DNA was seperated by DNA gel electrophoresis and stained with ethidium bromide subsequently. Typical DNA laddering indicative for apoptosis was visualized by UV light illumination.

In order to distinguish the mode of cell death induced by FWGE we treated a representative panel of human cancer cell lines with an IC_90 _of FWGE for 48 h. Subsequent to treatment, floating cells were harvested and an DNA gel electrophoresis was performed. Clearly, in all treated cell lines the typical 180 bp DNA laddering structure indicative for specific DNA degradation during the process of apoptosis could be detected (Figure 2).

### Combination of FWGE with 5-FU, Oxaliplatin and Irinotecan in human colon cancer cell lines

**Figure 3:**
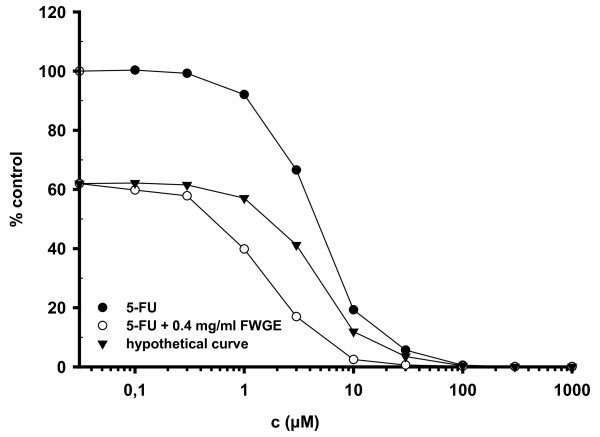
**Synergy between FWGE and 5-FU in human colon cancer cell line HCT15**. Plots represent the average of 3 independent experiments. The hypothetical curve was calculated as described by Drewinko et al. [16]. Synergy is indicated by the hypothetical curve which runs above the combination curve.

The combined drug effect of a parallel exposure to FWGE and either 5-FU, irinotecan or oxaliplatin was assessed in a panel of 8 colon cancer cell lines. The mode of drug interaction was analyzed by the method of Drewinko and the data summarized in table 1. Overall, mainly significant synergy was observed for the combinations of FWGE and 5-FU (6 out of 8 cell lines) and to a lesser extend for irinotecan and oxaliplatin (2 out of 8 cell lines). Drug interaction for the remaining cell lines was additive. Importantly, no significant antagonism was found for simultaneous drug exposure. A representative plot for synergistic drug interaction is presented in Figure 3.

**Table 1:** Summary of drug combinations

	IC50 (μM)								
**Cell line**	**Oxaliplatin ± FWGE**		**p-value**	**5-FU ± FWGE**		**p-value**	**CPT-11 ± FWGE**		**p-value**
	**-**	**+**		**-**	**+**		**-**	**+**	

HCT-8	0,43 ± 0,03	0,45 ± 0,03	0,52	2,65 ± 0,35	1,2 ± 0,6	0,023*	2,0 ± 0,46	1,8 ± 0,32	0,63
HCT-15	0,95 ± 0,19	0,57 ± 0,25	0,05	4,45 ± 0,72	1,45 ± 0,61	0,0001*	4,5 ± 0,3	3,4 ± 0,31	0,001*
HCT116	0,39 ± 0,06	0,19 ± 0,09	0,01*	4,6 ± 0,38	2,9 ± 0,9	0,01*	1,2 ± 0,1	0,96 ± 0,11	0,01*
HT29	0,32 ± 0,09	0,35 ± 0,05	0,53	0,99 ± 0,31	1,3 ± 0,6	0,39	3,5 ± 0,3	4,1 ± 0,23	0,05
DLD-1	2,47 ± 0,17	2,2 ± 0,8	0,61	3,2 ± 0,21	1,6 ± 0,7	0,02*	6,6 ± 0,6	6,1 ± 0,85	0,43
Colo205	0,45 ± 0,05	0,24 ± 0,05	0,001*	0,54 ± 0,12	0,44 ± 0,1	0,26	1,2 ± 0,19	1,1 ± 0,19	0,24
Colo320	1,1 ± 0,34	0,84 ± 0,13	0,33	1,35 ± 0,133	0,57 ± 0,03	0,001*	8,5 ± 3,4	8,7 ± 3,1	0,92
SW48	0,13 ± 0,02	0,1 ± 0,02	0,09	3,4 ± 0,2	2,2 ± 0,2	0,002*	2,4 ± 0,35	2,1 ± 0,29	0,18
SW480	0,57 ± 0,11	0,37 ± 0,12	0,06	2,7 ± 0,17	2,9 ± 1,5	0,83	6,4 ± 1,2	6,9 ± 2,3	0,72

n ≥ 3, asterisk indicates significant synergistic drug interaction

### Sequential drug application of FWGE and 5-FU in the human colon cancer cell lines HT29 and HCT-8

To evaluate the influence of drug scheduling, exponentially growing cells were exposed to an IC_30 _of FWGE 24 h after seeding which was followed by serial dilutions of 5-FU after further 24 hours or vice versa. Cells were fixated after 120 h total assay time and processed according to the SRB protocol. IC_50 _values were calculated based on the Hill equation using Sigma plot and the data were summarized in table 2. In both cell lines, if 5-FU was followed by FWGE, we observed an additive drug interaction. On the other hand, if FWGE precedes 5-FU for 24 hours, we observed a trend to antagonism in both cell lines. However, this antagonism did not reach statistical significance. Taken together, these findings suggest that the interactions between 5-FU and FWGE are schedule-dependent. Schedules in which FWGE precedes 5-FU should be avoided.

**Table 2:** Schedule effect of FWGE and 5-FU

	IC_50 _(μM)					
**Cell line**	**5-FU**	**5-FU→FWGE**	**p-value**	**5-FU**	**FWGE→5-FU**	**p-value**
HCT-8	1,52	1,57	> 0.05	1,74	2,20	> 0.05
HT29	1,10	1,06	> 0.05	1,77	2,23	> 0.05

n ≥ 3; cells were exposed to either 5-FU 24 h after plating followed by FWGE after additional 24 h or vice versa up to a total assay time of 120 h.

## Discussion

FWGE belongs to the group of nutraceuticals that are approved as dietary food for special medical purposes for cancer patients. It is well tolerated at the recommended doses and possesses a broad therapeutic window [2]. Beside its use as nutrition supplement to ameliorate cancer symptoms in patients there is incremental evidence that FWGE might exert some anticancer properties as well [1-3]. However, up to now this antitumor effect is only sparsely investigated.

Thus, we screened the preclinical cytotoxic activity of FWGE as a single agent or in combination with the commonly used cytostatics 5-FU, oxaliplatin or irinotecan in a large panel of human tumor cell lines to evaluate its potential antitumor properties.

Human tumor cell lines or human tumor xenografts commonly serve as models for preclinical drug screening. Still, care has to be taken in the interpretation of results since their positive predictive value is limited to approximately 60-70% [18,19]. The predictive value of preclinical cytotoxicity data could by strengthened by the model of relative antitumor activity. It allows to estimate the potential activity of a drug in a certain tumor type by taking the preclinical IC_50 _value and clinically achievable peak plasma concentrations into account [20]. Only if the preclinical IC_50 _value is clearly below the plasma concentration that can be achieved in a patient one can assume potential clinical activity.

In the present study we observed a significant antiproliferative activity of FWGE as assessed by IC_50 _concentrations which were in a similar range as reported by other investigators [7,8,21]. With a RAA ranging from approximately 1 to 24, FWGE appeared to have potential clinical activity in the broad spectrum of tumor entities used in our cell line screen. The highest activity was found in neuroblastoma and ovarian cancer cell lines. Of particular interest for further clinical development is the relative homogeneous sensitivity of the eight colon cancer cell lines employed in this study with IC_50 _values ranging from 0.3-0.54 mg/ml. This prompted us to perform combination experiments of FWGE and chemotherapy in the colon cancer model. Overall, we could demonstrate additive to synergistic drug interaction of FWGE with irinotecan, oxaliplatin and 5-FU. These data are in line with a previous clinical report of Jakab et al.. They observed in their study with colon cancer patients an increased survival rate and reduced development of metastasis for the combination of FWGE and 5-FU-based regimens [13]. However, their clinical trial is hampered by methodological limitations and thus, data from that study are of limited significance [1]. Regimens of 5-FU and folinic acid in combination with either oxaliplatin or irinotecan are the cornerstones in the adjuvant and/or palliative treatment of colorectal cancer today [22]. Therefore, the observed additive to synergistic effects and even more, the exclusion of antagonistic drug interaction in our colon cancer model is of pivotal relevance and provides the rationale for a potential combination of FWGE and irinotecan or oxaliplatin based treatment regimens in well designed randomized clinical trials.

The efficiency of drug combinations is often sequence dependent. In our cell line system we observed additive to synergistic drug interaction for parallel drug combinations of 5-FU and FWGE. These data confirm the results of Szende et al, who observed no decrease in the antiproliferative activity of 5-FU, doxorubicin or navelbine by the simultaneous exposure to nontoxic concentrations of FWGE [23].

In drug sequence experiments the additive to synergistic effect was abolished dependent on the sequence resulting in either additive effects or even a trend to antagonism (table 2). FWGE is known to interfere with ribonucleotide reductase which catalyzes the reduction of ribonucleotides to their corresponding deoxyribonucleotides [11]. Since these are the building blocks for DNA replication, pretreatment of cells with FWGE decreases DNA-synthesis which might hamper the activity of the antimetabolite 5-FU. In line with this hypothesis, it was recently demonstrated in HT29 and HL-60 cells, that pretreatment of cells with FWGE significantly reduced the deoxyribonucleotide triphosphate pools and the incorporation of ^14^C-cytidine into DNA [3,8]. In the event of impaired DNA-synthesis 5-FU might lose one of its targets which might at least in part explain the observed trend to antagonism in our model system when FWGE treatment precedes 5-FU by 24 hours. Taken together, for further development of drug combinations with FWGE not just the combination partner but also the chosen drug schedule appeared to be crucial and should be considered.

Based on its documented preclinical activity profile and mechanisms of drug action as well as on the available clinical data, FWGE appeared to be a good combination partner for drug regimens, in particular as modulator of drug activity and attenuator of drug toxicity.

In conclusion, FWGE exerted significant antiproliferative activity in a broad spectrum of tumor cell lines. Simultaneous administration of FWGE with 5-FU, oxaliplatin or irinotecan did not impair the cytotoxic activity of these cytostatic drugs in our colon cancer model. Our findings suggest that simultaneous application of 5-FU and FWGE, which resulted in additive to synergistic drug interactions, seems superior to sequential scheduling. The sequential administration of 5-FU followed by FWGE may be appropriate, while the reverse sequence should be avoided.

Overall, based on its preclinical activity profile and clinical available data, further evaluation of combinations FWGE and conventional cytostatic drugs seems safe and warranted.

## Abbreviations

FWGE: Fermented wheat germ extract; FBS: Fetal bovine serum; SRB: Sulforhodamine B; RAA: Relative antitumor activity; TCA: Trichloroacetic acid; FDA: Food and Drug Administration: 5-FU: 5-fluorouracil: DTIC: Dacarbazine; CPT-11: Irinotecan; PARP: Poly(ADP-ribose) polymerase

## Competing interests

The authors declare that they have no competing interests.

## Authors' contribution

TM carried out the cell line studies and contributed significantly to the design of the study. KJ performed the data analysis and preparation of figures. WV participated in the design of the study and data analysis. He prepared the manuscript and raised funding.

All authors read and approved the final manuscript.

## References

[B1] Telekes A Hegedus M Chae CH Vekey K Avemar (wheat germ extract) in cancer prevention and treatment Nutr Cancer 2009 61 891 899 10.1080/01635580903285114 20155632

[B2] Johanning GL Wang-Johanning F Efficacy of a medical nutriment in the treatment of cancer Altern Ther Health Med 2007 13 56 63 quiz 64-55 17405680

[B3] Illmer C Madlener S Horvath Z Saiko P Losert A Herbacek I Grusch M Krupitza G Fritzer-Szekeres M Szekeres T Immunologic and biochemical effects of the fermented wheat germ extract Avemar Exp Biol Med (Maywood) 2005 230 144 149 15673563 10.1177/153537020523000209

[B4] Fajka-Boja R Hidvegi M Shoenfeld Y Ion G Demydenko D Tomoskozi-Farkas R Vizler C Telekes A Resetar A Monostori E Fermented wheat germ extract induces apoptosis and downregulation of major histocompatibility complex class I proteins in tumor T and B cell lines Int J Oncol 2002 20 563 570 11836569

[B5] Hidvegi M Raso E Tomoskozi-Farkas R Paku S Lapis K Szende B Effect of Avemar and Avemar + vitamin C on tumor growth and metastasis in experimental animals Anticancer Res 1998 18 2353 2358 9703878

[B6] Boros LG Nichelatti M Shoenfeld Y Fermented wheat germ extract (Avemar) in the treatment of cancer and autoimmune diseases Ann N Y Acad Sci 2005 1051 529 542 10.1196/annals.1361.097 16126993

[B7] Comin-Anduix B Boros LG Marin S Boren J Callol-Massot C Centelles JJ Torres JL Agell N Bassilian S Cascante M Fermented wheat germ extract inhibits glycolysis/pentose cycle enzymes and induces apoptosis through poly(ADP-ribose) polymerase activation in Jurkat T-cell leukemia tumor cells J Biol Chem 2002 277 46408 46414 10.1074/jbc.M206150200 12351627

[B8] Saiko P Ozsvar-Kozma M Madlener S Bernhaus A Lackner A Grusch M Horvath Z Krupitza G Jaeger W Ammer K Fritzer-Szekeres M Szekeres T Avemar, a nontoxic fermented wheat germ extract, induces apoptosis and inhibits ribonucleotide reductase in human HL-60 promyelocytic leukemia cells Cancer Lett 2007 250 323 328 10.1016/j.canlet.2006.10.018 17137710

[B9] Boros LG Cascante M Lee WN Metabolic profiling of cell growth and death in cancer: applications in drug discovery Drug Discov Today 2002 7 364 372 10.1016/S1359-6446(02)02179-7 11893545

[B10] Boros LG Lapis K Szende B Tomoskozi-Farkas R Balogh A Boren J Marin S Cascante M Hidvegi M Wheat germ extract decreases glucose uptake and RNA ribose formation but increases fatty acid synthesis in MIA pancreatic adenocarcinoma cells Pancreas 2001 23 141 147 10.1097/00006676-200108000-00004 11484916

[B11] Shao J Zhou B Chu B Yen Y Ribonucleotide reductase inhibitors and future drug design Curr Cancer Drug Targets 2006 6 409 431 10.2174/156800906777723949 16918309

[B12] Demidov LV Manziuk LV Kharkevitch GY Pirogova NA Artamonova EV Adjuvant fermented wheat germ extract (Avemar) nutraceutical improves survival of high-risk skin melanoma patients: a randomized, pilot, phase II clinical study with a 7-year follow-up Cancer Biother Radiopharm 2008 23 477 482 10.1089/cbr.2008.0486 18771352

[B13] Jakab F Shoenfeld Y Balogh A Nichelatti M Hoffmann A Kahan Z Lapis K Mayer A Sapy P Szentpetery F Telekes A Thurzo L Vagvolgyi A Hidvegi M A medical nutriment has supportive value in the treatment of colorectal cancer Br J Cancer 2003 89 465 469 10.1038/sj.bjc.6601153 12888813 PMC2394381

[B14] Pfeiffer B Preiß J Unger C Avemar 2006 Onkologie integrativ, Urban & Fischer Verlag München 226 229

[B15] Skehan P Storeng R Scudiero D Monks A McMahon J Vistica D Warren JT Bokesch H Kenney S Boyd MR New colorimetric cytotoxicity assay for anticancer-drug screening J Natl Cancer Inst 1990 82 1107 1112 10.1093/jnci/82.13.1107 2359136

[B16] Drewinko B Dipasquale MA Yang LY Barlogie B Trujillo JM The synergistic lethal interaction of cis-diamminedichloroplatinum and natural nucleosides is related to increased DNA cross-links Chem Biol Interact 1985 55 1 12 10.1016/S0009-2797(85)80116-2 4064187

[B17] Ohe Y Nakagawa K Fujiwara Y Sasaki Y Minato K Bungo M Niimi S Horichi N Fukuda M Saijo N In vitro evaluation of the new anticancer agents KT6149, MX-2, SM5887, menogaril, and liblomycin using cisplatin- or adriamycin-resistant human cancer cell lines Cancer Res 1989 49 4098 4102 2472873

[B18] Berger DP Henss H Winterhalter BR Fiebig HH The clonogenic assay with human tumor xenografts: evaluation, predictive value and application for drug screening Ann Oncol 1990 1 333 341 2261375 10.1093/oxfordjournals.annonc.a057770

[B19] Schroyens W Tueni E Dodion P Bodecker R Stoessel F Klastersky J Validation of clinical predictive value of in vitro colorimetric chemosensitivity assay in head and neck cancer Eur J Cancer 1990 26 834 838 10.1016/0277-5379(90)90165-P 2145907

[B20] Voigt W Bulankin A Muller T Schoeber C Grothey A Hoang-Vu C Schmoll HJ Schedule-dependent antagonism of gemcitabine and cisplatin in human anaplastic thyroid cancer cell lines Clin Cancer Res 2000 6 2087 2093 10815936

[B21] Marcsek Z Kocsis Z Jakab M Szende B Tompa A The efficacy of tamoxifen in estrogen receptor-positive breast cancer cells is enhanced by a medical nutriment Cancer Biother Radiopharm 2004 19 746 753 10.1089/cbr.2004.19.746 15665622

[B22] Labianca R Nordlinger B Beretta GD Brouquet A Cervantes A Primary colon cancer: ESMO Clinical Practice Guidelines for diagnosis, adjuvant treatment and follow-up Ann Oncol 21 Suppl 5 v70 77 20555107 10.1093/annonc/mdq168

[B23] Szende B Marcsek Z Kocsis Z Tompa A Effect of simultaneous administration of Avemar and cytostatic drugs on viability of cell cultures, growth of experimental tumors, and survival tumor-bearing mice Cancer Biother Radiopharm 2004 19 343 349 10.1089/1084978041425016 15285880

